# Identification of ferroptosis/autophagy-related genes and potential underlying mechanisms involved in the effect of BMSC senescence on the osteogenic differentiation of aging BMSCs

**DOI:** 10.1016/j.gendis.2024.101259

**Published:** 2024-03-08

**Authors:** Kai Chen, Huaqiang Tao, Haixiang Xiao, Miao Chu, Pengfei Zhu, Shujun Lv, Lixin Huang, Dechun Geng

**Affiliations:** a Department of Orthopedics, Hai'an People's Hospital, Hai'an, Jiangsu 226600, China; b Department of Orthopedics, The First Affiliated Hospital of Soochow University, Suzhou, Jiangsu 215000, China; c Department of Orthopaedics, Dushu Lake Hospital Affiliated to Soochow University, Suzhou, Jiangsu 215000, China; d Department of Orthopaedics, Yixing People's Hospital, Yixing, Jiangsu 214200, China

Bone mesenchymal stem cells (BMSCs) are stem cells located in the bone marrow matrix that have a variety of differentiation potentials and biological functions. They play an important role in bone regenerative medicine. The senescence of BMSCs might cause accelerated degeneration of bone tissue. Autophagy is a process in which cellular homeostasis is maintained by autophagosomes and lysosomes. It could control the function and senescence of BMSCs during bone aging and might be a therapeutic target for treating diseases during aging.^1^ Ferroptosis is a regulated cell death process.^2^ The inhibition of ferroptosis in mesenchymal stem cells could reduce cell injury and might have great therapeutic value.^3^, ^4^, ^5^

Autophagy and ferroptosis might both play important roles during the senescence of BMSCs. To determine whether ferroptosis- or autophagy-related genes are involved in the regulation of osteogenic differentiation in aging BMSCs, bioinformatics methods were applied in this study (Fig. S1, drawn from the BioRender network, https://biorender.com/). Three datasets, GSE35957, GSE135401, and GSE148049 (osteogenic differentiation), were obtained from the Gene Expression Omnibus. The differentially expressed genes (DEGs) and abnormally expressed miRNAs were analyzed via GEO2R. Ferroptosis- and autophagy-related genes were downloaded from relevant databases. The target genes of the miRNAs affecting osteogenesis were evaluated via FunRich software. Functional and pathway enrichment were explored via the Metascape and FunRich software. The String database and Cytoscape software were used to construct a protein–protein interaction network and identify hub genes. The GSE154748 (including two mouse models of osteogenesis imperfecta) and single-cell sequencing dataset GSE147287 were used as verification datasets. CTDbase, Network Analyst, the HPA database, and the GeneAnalytics database were used to research the functions, disease relationships, tissue and cell expression, regulatory effects, interactions, and pathways associated with significant hub genes. Additionally, *in vitro* experiments, such as reverse transcription PCR, Alizarin red staining, and immunofluorescence analysis, were performed for verification. The primers used to amplify the hub genes are listed in Table S1.

The volcano plots in Figure S2 show the DEGs obtained from GEO2R. The box plots showed a satisfactory normalization level. We set different |log2 fold change (FC)| levels depending on the researchers' reports of the datasets (|log2 FC| > 1 in GSE35957, |log2 FC| > 0.32 in GSE135401, and |log2 FC| > 0.58 in GSE148049). The heatmaps of the top 50 DEGs (25 up-regulated plus 25 down-regulated genes) are listed in Figure S2E and F. In total, 591 ferroptosis-related genes and 293 autophagy-related genes were downloaded from the FerrDB, HaDB, and ATDB databases. The intersections among ferroptosis, autophagy, GSE35957, and GSE135401 are shown in the Venn diagrams of Figure 1A and B. In GSE35957, 89 (44 + 7 + 27 + 9 + 2) DEGs were identified. However, in GSE135401, 28 (9 + 2 + 11 + 1 + 5) DEGs were identified. The 11 (9 + 2) DEGs between GSE35957 and GSE135401 were also analyzed. Eight (5 up-regulated plus 3 down-regulated) DEGs with the same expression trend were identified (Fig. 1B). The uncrossed DEGs in the two datasets (44 + 7 + 27 in GSE35957; 11 + 1 + 5 in GSE135401) were also collected, and a heatmap was drawn (Fig. S3A, B). Finally, 103 DEGs (44 + 7 + 27 + 11 + 1 + 5 + 5 + 3) were identified for further study. GO and KEGG enrichment analyses of the 103 DEGs were performed via the Metascape database. The results are visualized in Figure 1C, D and Figure S3C, D. The most common GO pathways included autophagy and positive regulation of cell death. The most common KEGG pathways were involved in autophagy-animal and pathways in cancer.

**Figure 1:**
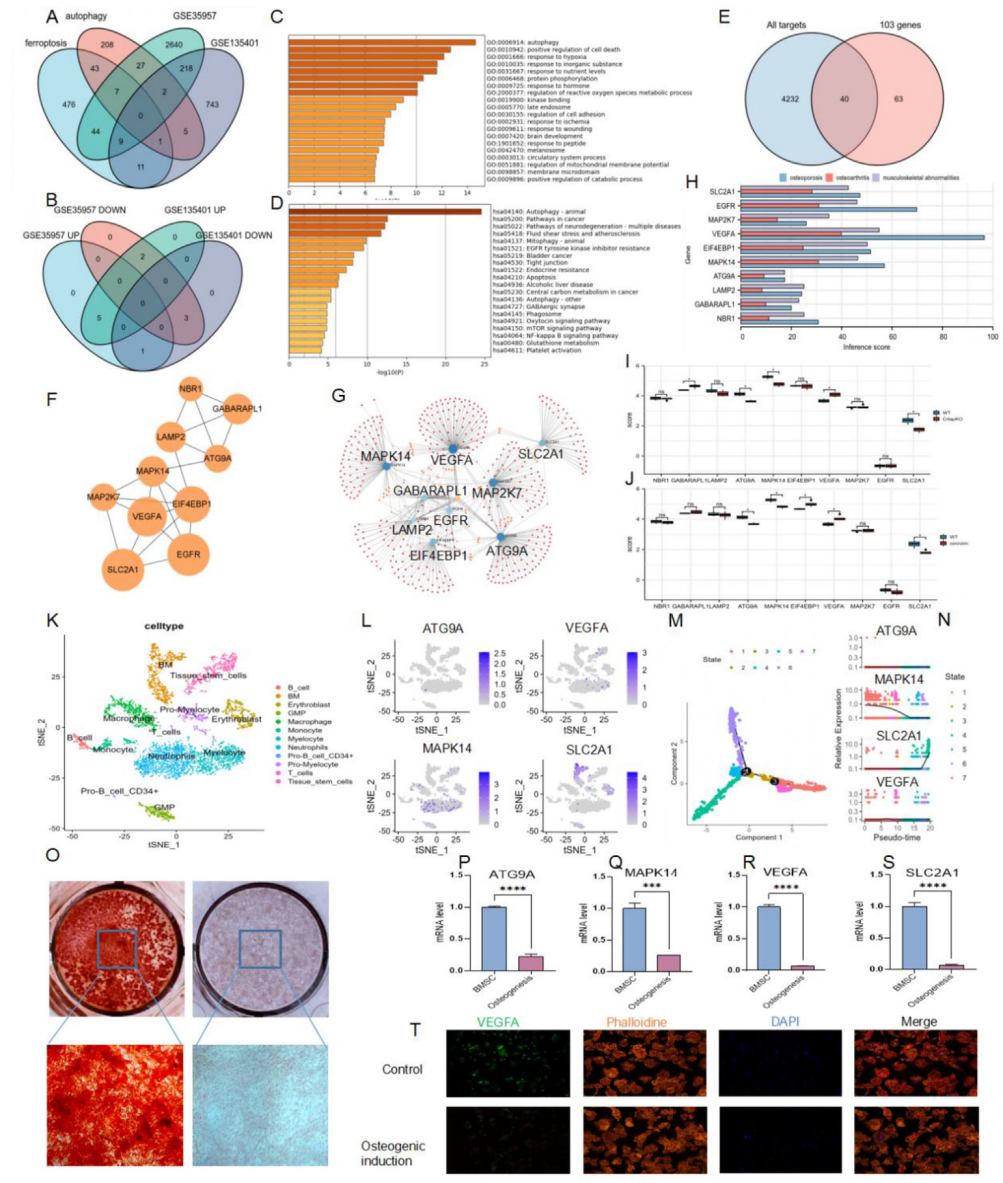
Results of the study. **(A)** Venn diagram of GSE35957, GSE135401, ferroptosis, and autophagy related genes. **(B)** Venn diagram of the 11 genes (9 + 2) screened from GSE35957 and GSE135401: 5 co-highly expressed genes and 3 co-low expressed genes were discovered. **(C)** GO enrichment results from Metascape network. **(D)** KEGG enrichment results from Metascape network. **(E)** Venn diagram of 4262 target mRNAs of the miRNAs (predicted by FunRich software) and the 103 genes from A (44 + 7 + 27 + 11 + 1 + 5) and B (5 + 3). **(F)** Top 10 hub genes identified by cytoHubba application via MCC method. **(G)** Normal and minimum gene-miRNA interaction network of the 10 hub genes. **(H)** Histogram of the inference scores from CTDbase. In this figure, the inference scores of the 10 hub genes on osteoporosis, osteoarthritis, and musculoskeletal abnormalities were listed. **(I, J)** Expression levels of the 10 hub genes in a mouse model of osteogenesis imperfecta. (I) Crtapko mod. (J) oim/oim modle. Only four genes had the same expression trend in both models (ATG9A, MAPK14, VEGFA, SLC2A1). The data type was FPKM. Wilcoxon rank-sum test was used. *P* value < 0.05 meant statistical significance. **(K–N)** Single-cell analysis results. (K) Cell types of osteoporosis and osteoarthritis samples. (L) The expression levels and cell distributions of ATG9A, VEGFA, MAPK14, and SLC2A1. (M) Branch and state of pseudotime analysis results. (N) Dynamic change of expression levels of the four genes. **(O–T)***In vitro* validation. (O) Alizarin red staining of BMSC during osteogenic induction (21 days). (P–S) PCR results of the four genes in BMSC during osteogenesis. The expression levels of ATG9A, VEGFA, MAPK14, and SLC2A1 were down-regulated during osteogenesis of BMSC cells. (T) Immunofluorescence of BMSC after osteogenic induced for three days.

A volcano plot and box plot of the miRNAs in GSE148049 are shown in Figure S4A and B. A total of 148 miRNAs were identified, analyzed, and enriched as shown by FunRich software (Fig. S4C–F). Finally, 4272 target genes of the 148 miRNAs were identified. Compared with the 103 DEGs, 40 osteogenic-related DEGs were ultimately identified (Fig. 1E and Table S2).

The protein–protein interaction network was analyzed via the STRING database and Cytoscape software (Fig. S5A). With the MCODE application, three subnetworks were identified (Fig. S5B–D). The top 10 hub genes, NBR1, GABARAPL1, LAMP2, ATG9A, MAPK14, EIF4EBP1, MAP2K7, VEGFA, EGFR, and SLC2A1, were identified via the cytoHubba application using the MCC method (Fig. 1F). The most common pathways associated with these 10 genes were involved in malignant pleural mesothelioma, the HIF-1 signaling pathway, and autophagy (Fig. S5E).

The NetworkAnalyst database was used to analyze the gene-miRNA and gene-disease networks (Fig. 1G; Fig. S6). The hub genes were related to many miRNAs and diseases. The inference scores from CTDbase for the 10 hub genes related to osteoporosis, osteoarthritis, and musculoskeletal abnormalities showed that these genes also play important roles in the progression of orthopedic diseases (Fig. 1H). For example, the inference score of VEGFA for osteoporosis was the highest among the 10 hub genes.

In the Crtapko mouse model, GABARAPL1, ATG9A, MAPK14, VEGFA, and SLC2A1 were significantly differentially expressed (Fig. 1I). However, for the oim/oim model, ATG9A, MAPK14, EIF4EBP1, VEGFA, and SLC2A1 were significant (Fig. 1J). Finally, we identified four significant genes affecting the imperfecta mouse model (ATG9A, MAPK14, VEGFA, and SLC2A1). These four genes might affect the progression of osteogenesis imperfecta disease. The miRNAs related to osteogenic progression (GSE148049) are shown in Table S2: ATG9A (hsa-miR-34c-5p; hsa-miR-15b-5p), VEGFA (hsa-miR-15b-5p), MAPK14 (hsa-miR-125b-5p; hsa-miR-125a-5p), and SLC2A1 (hsa-miR-148a-3p; hsa-miR-328-3p). We speculate that these four genes and relevant miRNAs might participate in the osteogenic progression of BMSCs. During the aging process, BMSCs might be involved in the regulation of cell senescence and might affect the osteogenic ability of old BMSCs. The immunohistochemistry and immunofluorescence results for the four genes significantly related to the HPA dataset are listed in Figure S7A. In Table S3, the top ten regulatory pathways of the four significant genes are listed. VEGFA was a member of all the top ten pathways. In addition, signaling network analysis of the four genes was performed via the NetworkAnalyst database, and the results are shown in Figure S6D.

For single-cell analysis, approximately 12 kinds of cells were identified from the samples of osteoporosis patients and osteoarthritis patients (Fig. 1K). The cell distributions of the four hub genes are listed in Figure 1L. The results of pseudotime analysis showed that the expression levels of these four genes changed dynamically during cell development (Fig. 1M and N). Additionally, the significant genes associated with each pseudotime state are listed as a heatmap in Figure S8A.

Figure 1O showed that the BMSCs we used had good osteogenic induction ability according to the Alizarin red staining results. The reverse transcription PCR results verified the down-regulation of ATG9A, MAPK14, VEGFA, and SLC2A1 during the osteogenic induction process of BMSCs (Fig. 1P–S). The immunofluorescence results further confirmed the downward trend in VEGFA expression (Fig. 1T).

In summary, our study revealed that ferroptosis-related genes (VEGFA, MAPK14, and SLC2A1) and autophagy-related genes (ATG9A and VEGFA) play important roles in BMSC senescence. These genes could affect the osteogenic differentiation ability of old BMSCs. The differential expression of these four genes is associated with osteogenesis imperfecta (in mouse models). These genes are related to osteoporosis and osteoarthritis and might be potential targets for reversing the senescence process and restoring the osteogenic differentiation ability of old BMSCs. However, their therapeutic value requires further study.

## Author contributions

KC, HT, and HX designed the research strategy. KC, HT, MC, and PZ worked with the *in vitro* experiments and performed reverse transcription PCR and immunofluorescence analysis. HX performed the Alizarin red staining. KC, SL, and LH performed the statistical analysis and wrote the manuscript. HT, LH, and DG revised the paper. All authors read and approved the final manuscript.

## Conflict of interests

The authors declare that they have no competing interests.

## Funding

This work was supported by the 10.13039/501100001809National Natural Science Foundation of China (No. 82072425), the 10.13039/501100004608Natural Science Foundation of Jiangsu Province, China (No. BK20220059), and the Jiangsu Medical Research Project (China) (No. ZD2022021).

## Data availability

The datasets generated and/or analyzed during the current study are available from the corresponding author upon reasonable request.
