# Supplementary material for: Identification of ferroptosis/autophagy-related genes and potential underlying mechanisms involved in the effect of BMSC senescence on the osteogenic differentiation of aging BMSCs
Source: Genes Dis. 2024 Mar 8;12(1):101259. doi: 10.1016/j.gendis.2024.101259 (PMC11530586; doi:10.1016/j.gendis.2024.101259)
Supplement: Multimedia component 1 [file mmc1.docx]

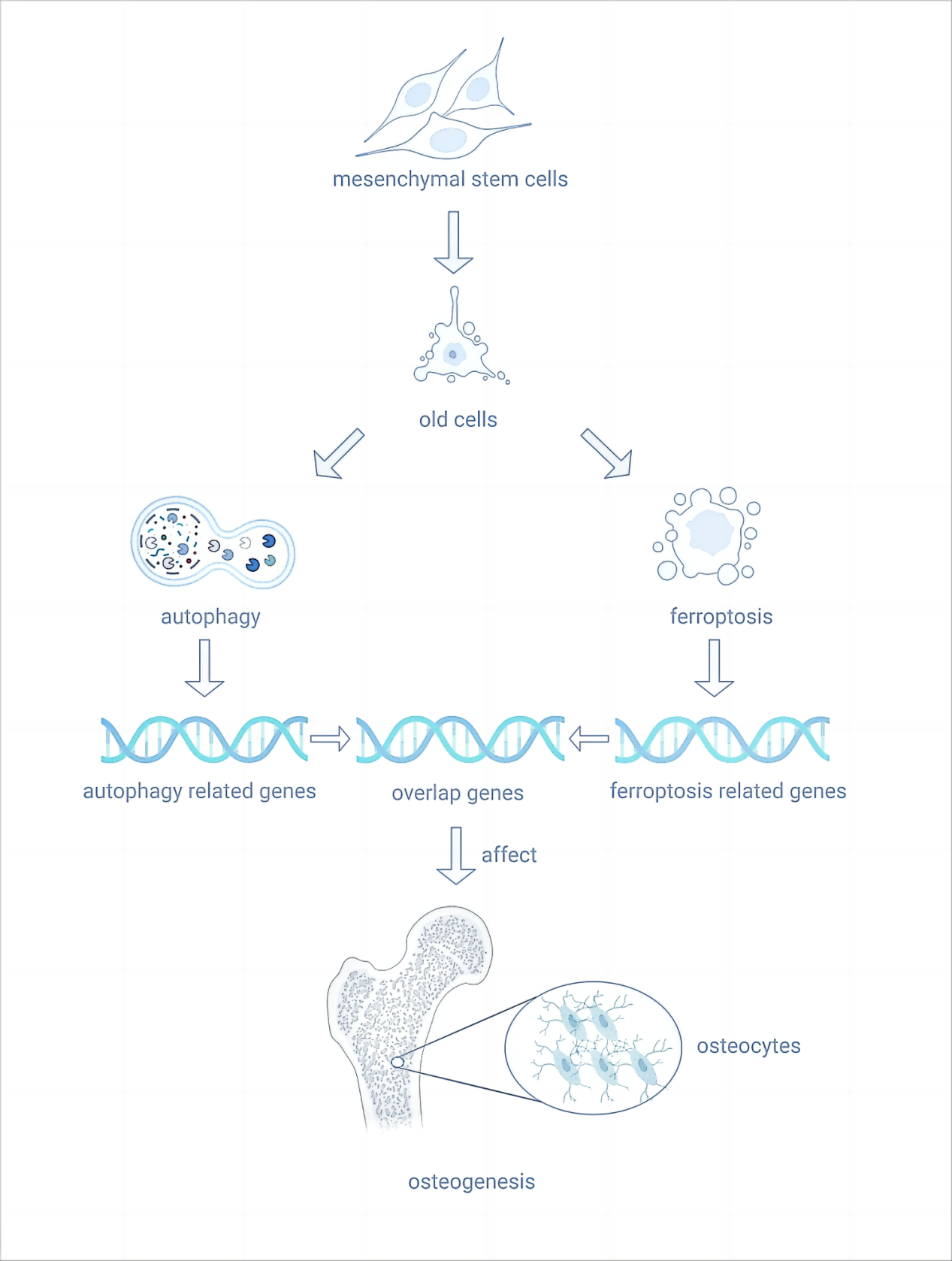


**Supplementary Figure 1** Sketch map of this study.

During bone mesenchymal stem cells (BMSC) senescence, we found a number of genes involved in autophagy and ferroptosis to be abnormally expressed. Further analysis revealed that these genes were also differentially expressed during cellular osteogenesis and were validated in a gene expression dataset from a mouse model of osteogenesis imperfecta. Drawed by biorender network (https://biorender.com/).


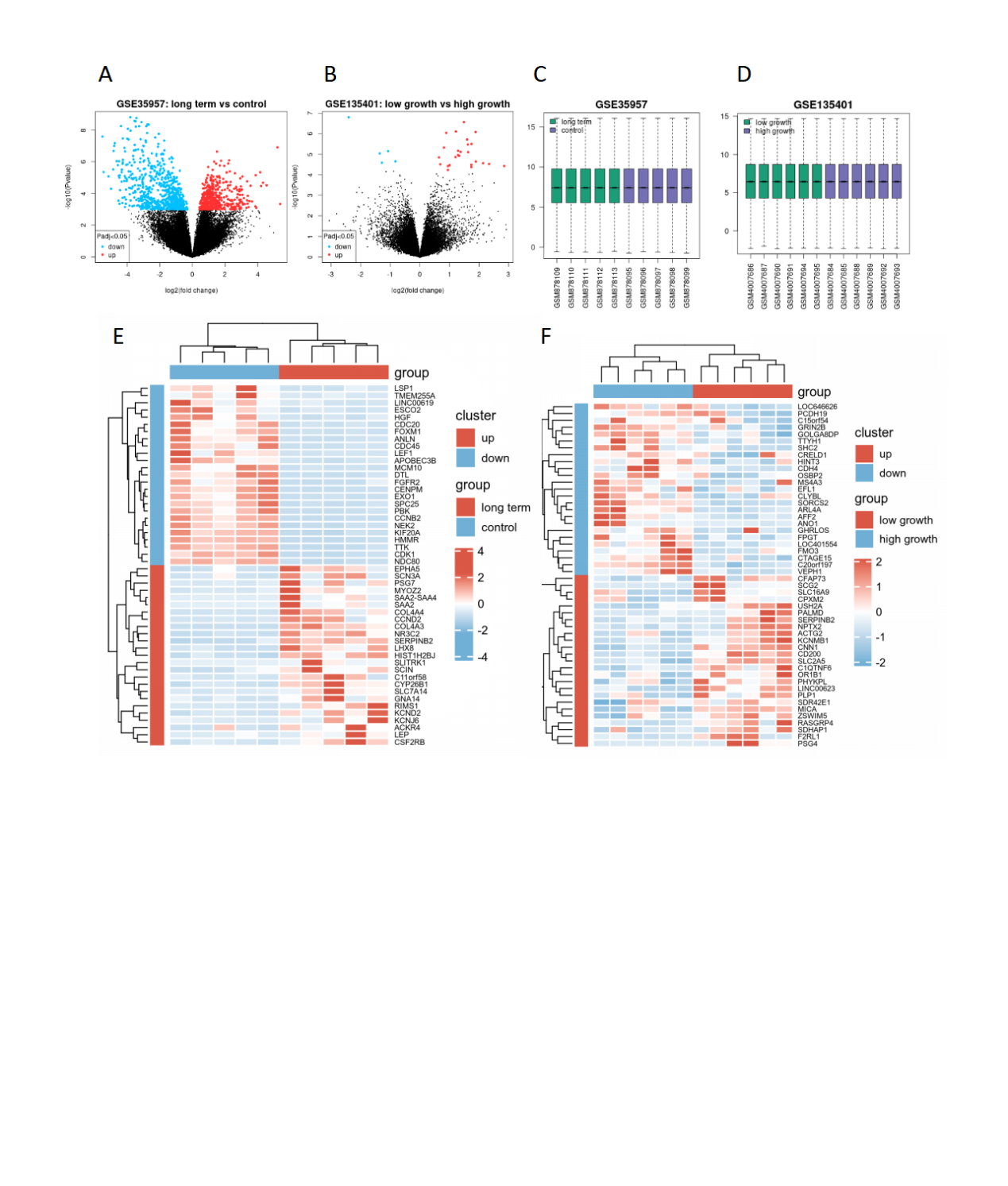
**Supplementary Figure 2** Analyze of the two gene sets.

**A:** volcano plot of GSE35957. **B:** volcano plot of GSE135401. **C:** box plot of GSE35957 (normalization). **D:** box plot of GSE135401 (normalization). **E:** top 50 genes of GSE35957. **F:** top 50 genes of GSE135401.


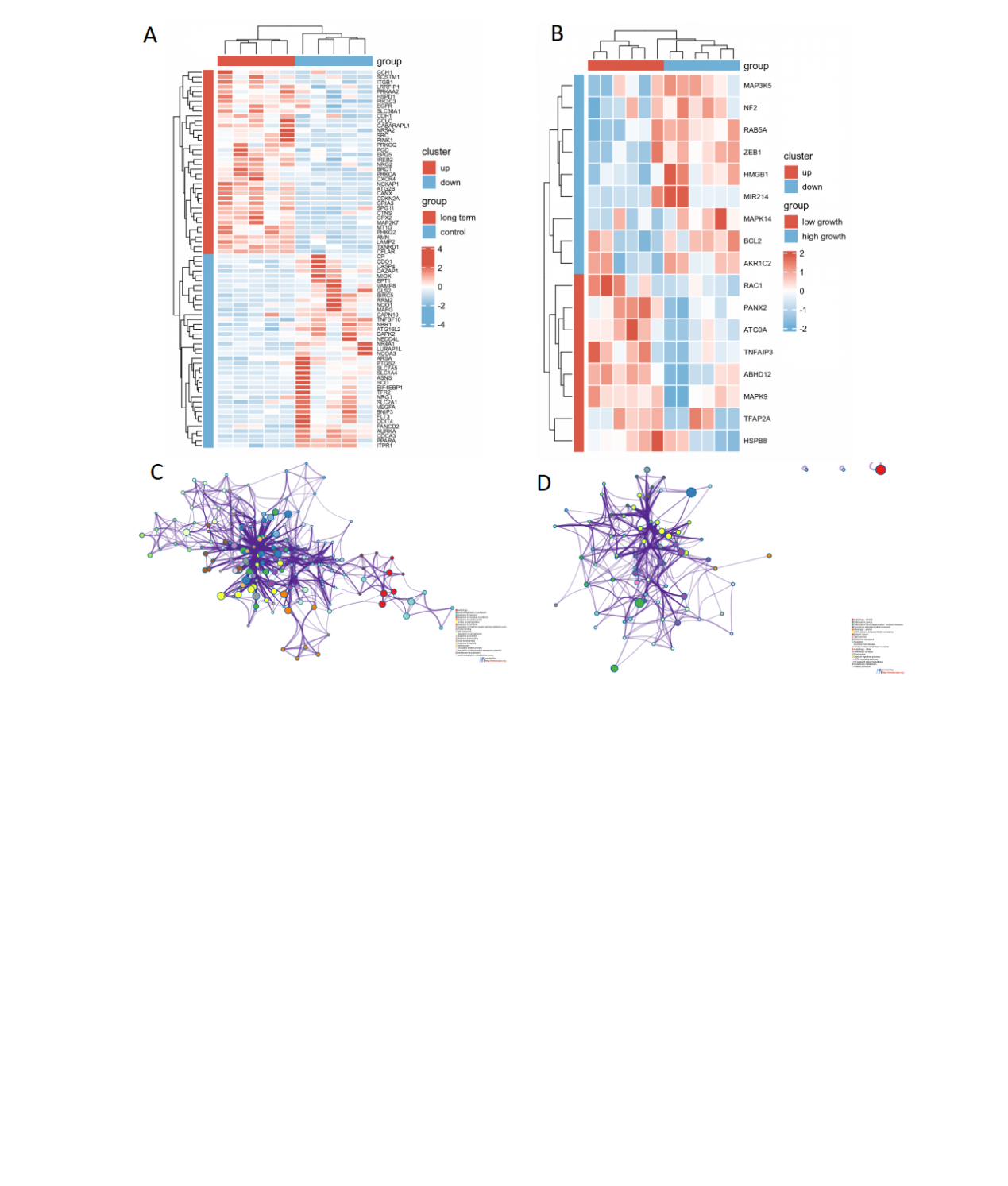


**Supplementary Figure 3** Heat maps and enrichment of screened genes.

**A:** Heat map of 78 genes from GSES5957 (44+7+27, not including the 9+2 genes in fig 4). **B:** Heat map of 17 genes from GSES5957 (11+1+5, not including the 9+2 genes in fig 4). **C:** GO enrichment results from Metascape network. **D:** KEGG enrichment results from Metascape network.


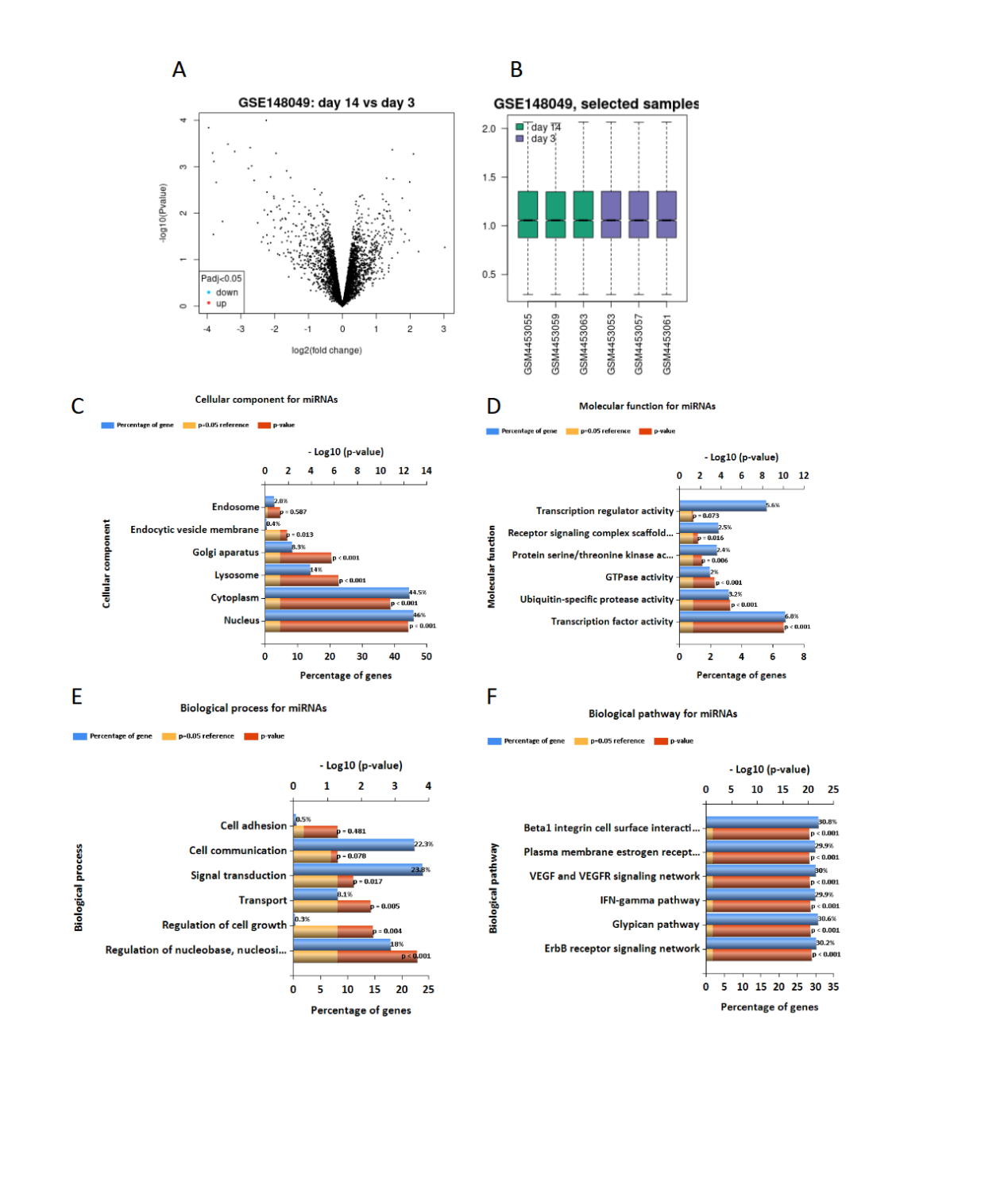


**Supplementary Figure 4** Analysis results of miRNAs in GSE148049.

**A, B:** Volcano and box plot from GEO2R. **C-F:** Enrichment results of the differentially expressed miRNAs from GSE148049 researched by FunRich software.


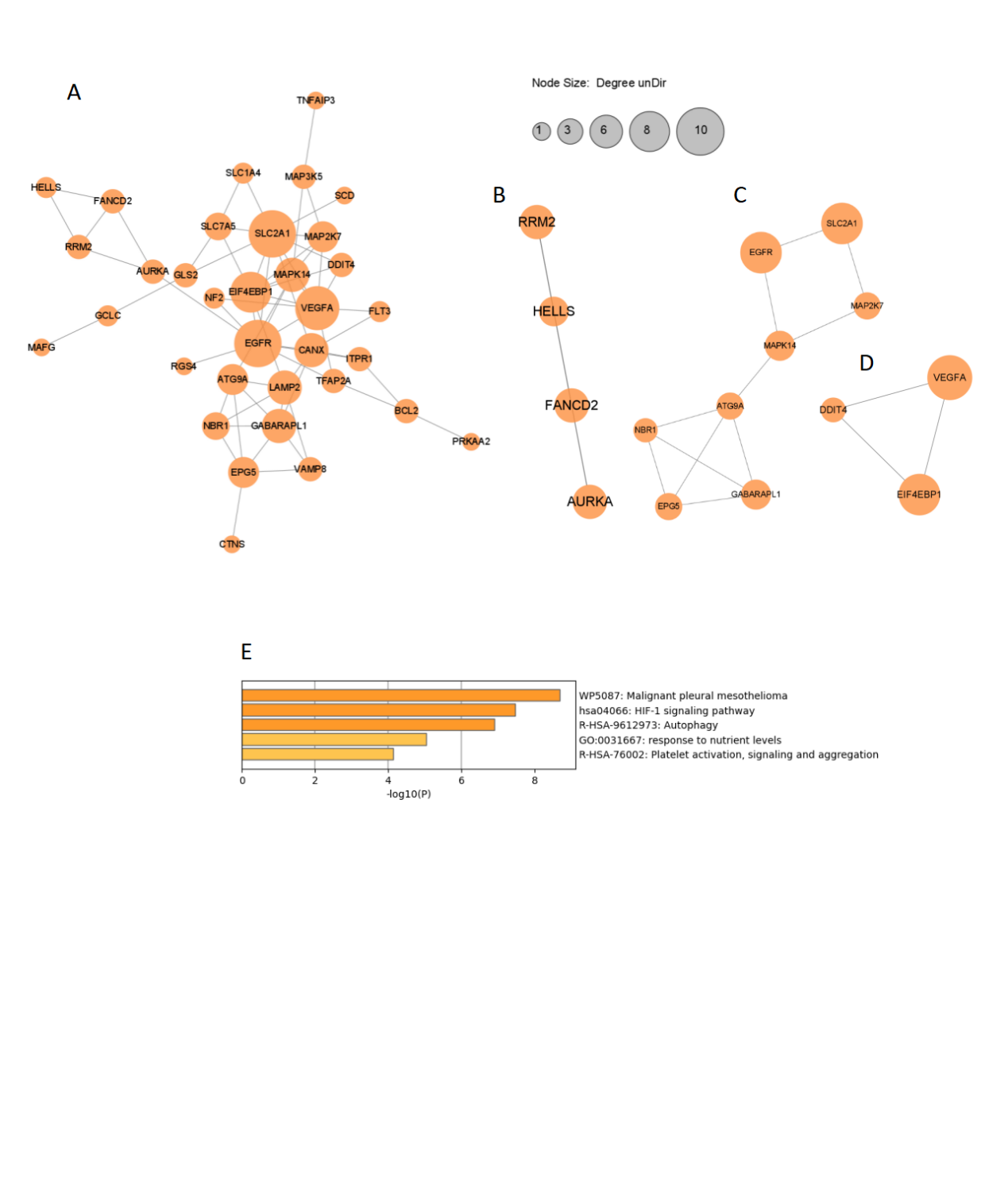


**Supplementary Figure 5** Protein-protein interaction (PPI) and hub genes analysis.

**A:** PPI network (downloaded from String network and draw by Cytoscape software). **B-D:** 3 sub networks identified by MCODE app. **E:** GO and KEGG enrichment of the 10 hub genes by Metascape network.


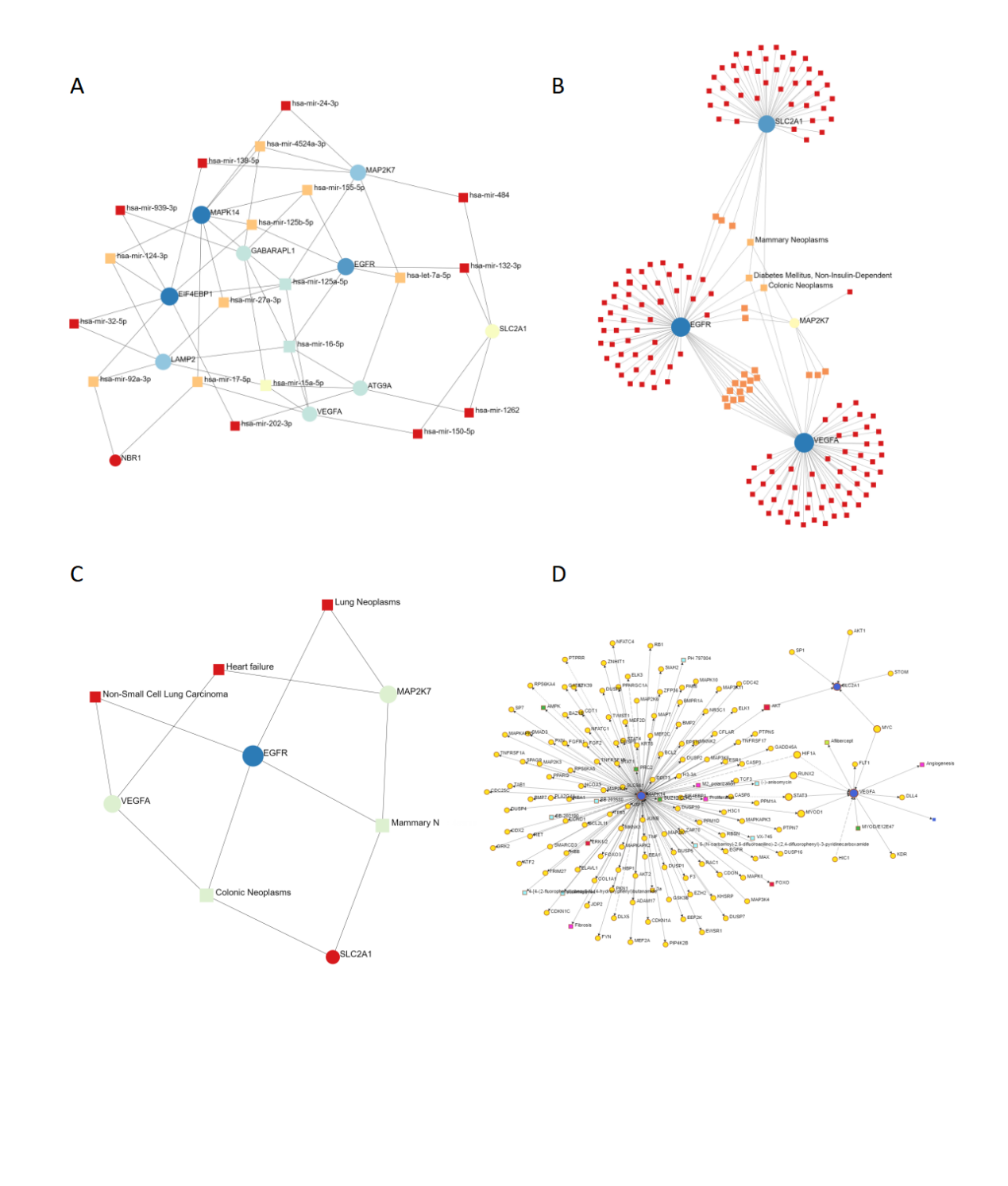


**Supplementary Figure 6** Networkanalyst results of the 10 hub genes.

**A:** Minimum gene-miRNA interaction network of the 10 hub genes. **B, C:** Normal and minimum gene-disease association network of the 10 hub genes. **D:** Signaling network analysis among the 4 genes identified by Networkanalyst database. There were relationships and regulation networks among MAPK14, SLC2A1 and VEGFA.


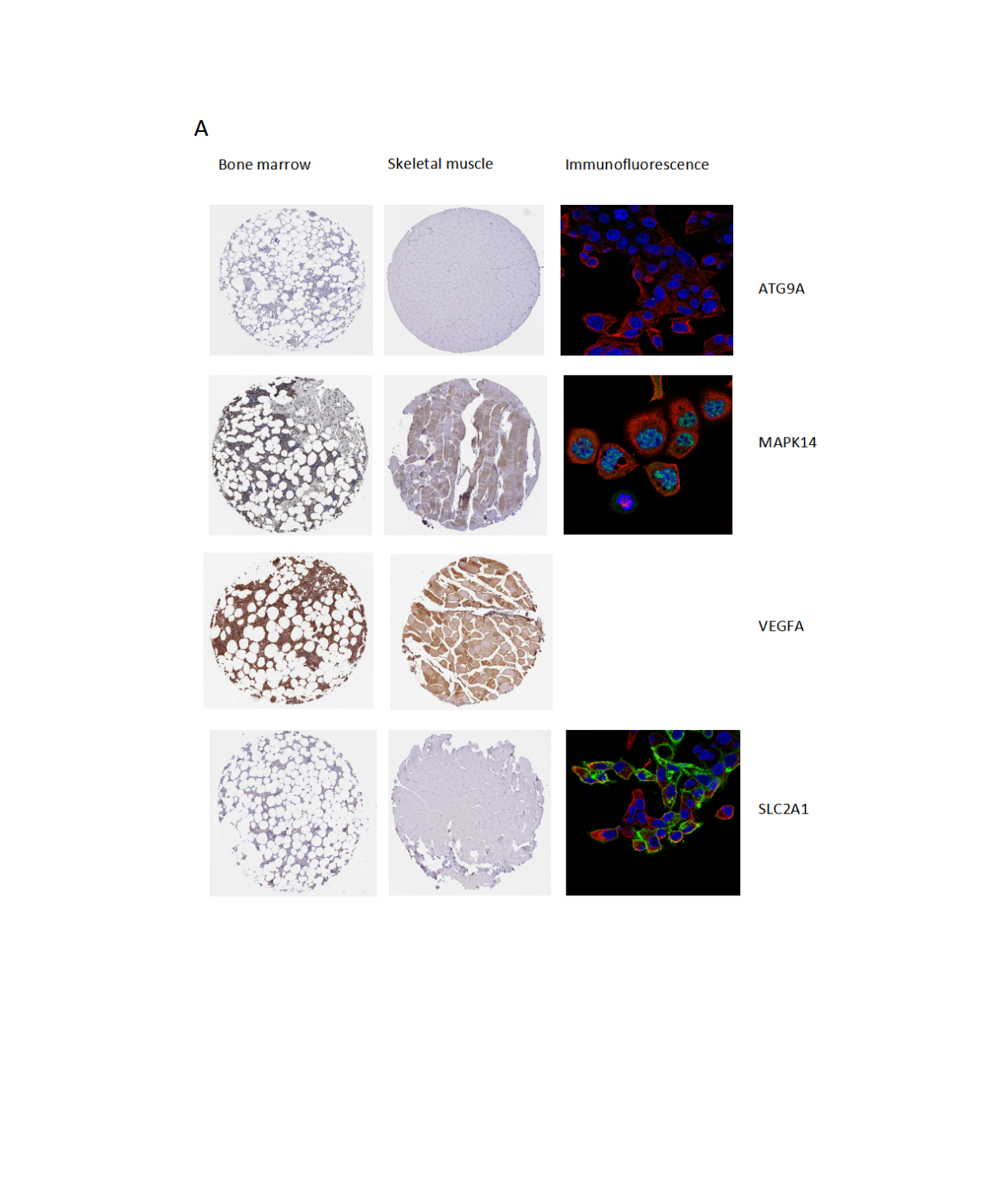


**Supplementary Figure 7** Validation by HPA databases.

**A:** Immunohistochemistry and immunofluorescence staining results from HPA database. Immunohistochemistry results of ATG9A in bone marrow and skeletal muscle were both negative. Immunofluorescence of ATG9A in Hep G2 cell showed that ATG9A was most expressed in the vesicles. Immunohistochemistry sections of MAPK14 in bone marrow and skeletal muscle were both positive. Immunofluorescence of MAPK14 in A-431 cell showed that MAPK14 was most expressed in the nuclear speckles and cytosol. Immunohistochemistry results of VEGFA in bone marrow and skeletal muscle were positive. Immunohistochemistry results of SLC2A1 in bone marrow and skeletal muscle were negative. Immunofluorescence of SLC2A1 in Hep G2 cell showed that SLC2A1 was most expressed in the plasma membrane.


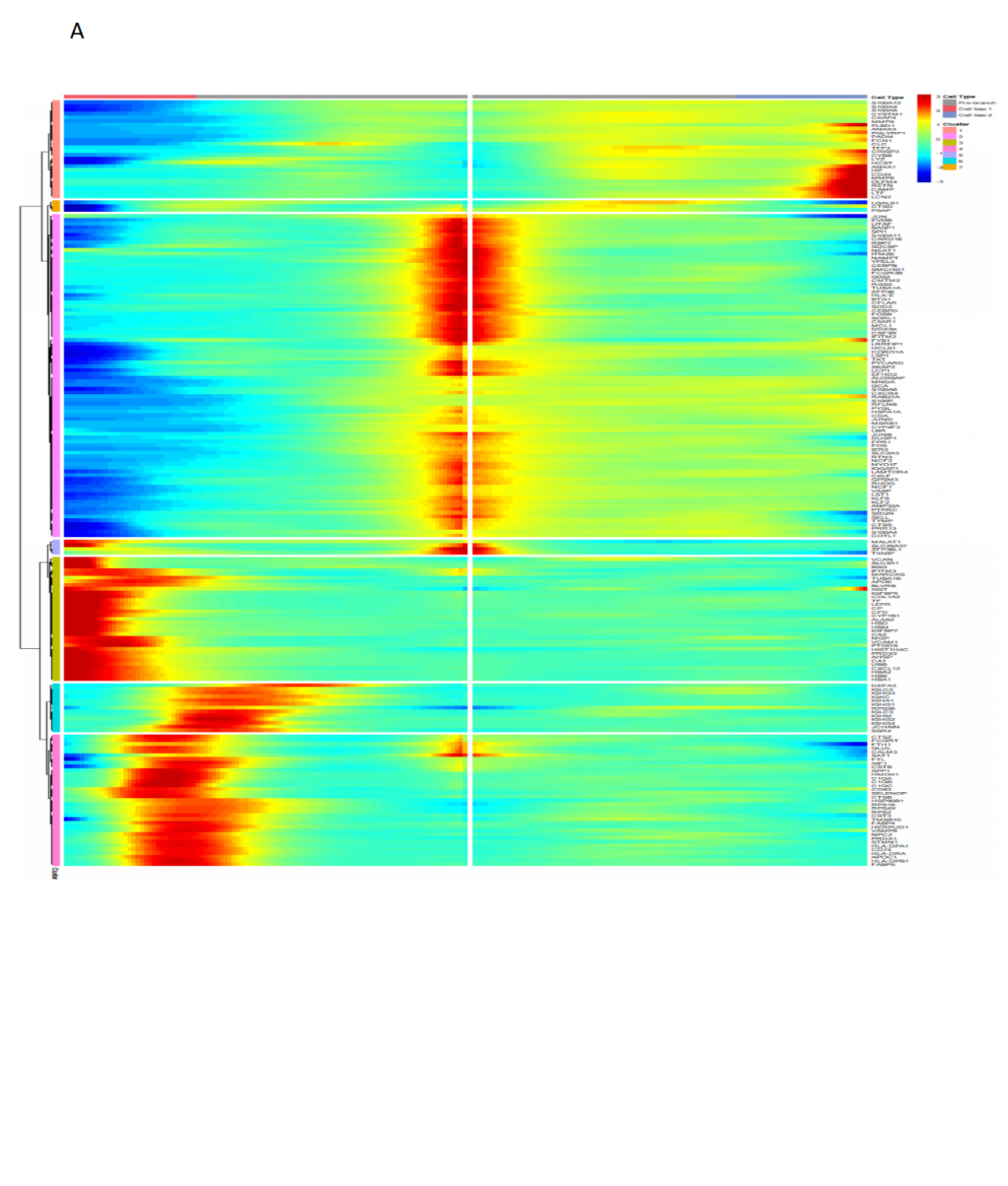


**Supplementary Figure 8** Single cell analysis results.

**A:** Heatmap of the significant genes of different states.

**Methods:**

***Public data retrieval and analyses***

Four datasets were downloaded from the GEO database (<https://www.ncbi.nlm.nih.gov/geo/>), including GSE35957 (platform GPL570, array), GSE135401 (platform GPL10558, microarray), GSE148049 (platform GPL21572， and GSE154748 (platform GPL19057, RNA sequencing, FPKM data format) . According to their references, GSE35957, GSE135401 and GSE148049 were analyzed by GEO2R tool (https://www.ncbi.nlm.nih.gov/geo/geo2r/) by different parameters. “limma precision weights” and “force normalization” were set in GEO2R. For GSE35957, 5 long-term cultivated (cellular senescence state) bone marrow mesenchymal stem cell (BMSC) samples and 5 control samples were selected. For differentially expressed genes (DEGs), the cut-off standard was P<0.05 and |log2 fold change (FC)|>1 [1]. For GSE135401, 6 low growth BMSC samples and 6 high growth samples were chosen. The cut-off standard was set as P<0.05 and |log2 FC|>0.32 (FC>1.25) as the researchers reported in their study [2]. And for GSE148049, we chose 3 samples at timepoint day 14 (long-term osteogenesis) and 3 at timepoint day 3 (short-term osteogenesis) during osteogenic differentiation of BMSC. And the cut-off standard was set as P<0.05 and |log2 FC|>0.58 (FC>1.5) as the researcher reported [3]. The analysis results, volcano plots and box plots were downloaded from GEO2R for further study. To find target genes of the miRNAs, the software FunRich v3.1.3 was used. At first, differently expressed miRNAs was imported in FunRich, and then “find targets” tool was used. R software (v4.2.0 <https://www.r-project.org/>) was utilized for data visualization by different packages (ggplot2 v3.3.6 for data visualization and Venn diagram drawing, ComplexHeatmap v2.12.0 for heat map drawing). And the box plot displaying expression levels of DEGs in GSE154784 (FPKM) was also draw by R software. And Wilcoxon rank sum test was used to statistically analyze the FPKM data, P value<0.05 meant significant.

***Autophagy and ferroptosis related genes***

Autophagy related genes were download from HaDB database (<http://www.autophagy.lu/>) and ATDB database ([http://www.bigzju.com/ATdb/#/Homepage](http://www.bigzju.com/ATdb/#/Homepage)). Duplicate genes were excluded. Ferroptosis related genes were obtained from FerrDB database (<http://www.zhounan.org/ferrdb/current/>). Venn diagram of autophagy related genes, ferroptosis related genes, differently expressed genes (DEGs) in GSE35957 and GSE135401 was draw by R software.

***GO and KEGG enrichment***

In order to perform Gene ontology (GO) and Kyoto Encyclopedia of Genes and Genomes (KEGG) pathway enrichment of the DEGs, the Metascape network was used (<https://metascape.org/gp>) [4]. “Min overlap: 3, P value cutoff: 0.01 and min enrichment: 1.5” were set as cut-off standard. The GO and biological pathway enrichment and result visualization of miRNAs in GSE148049 was carried out by FunRich software. “miRNA enrichment” tool of FunRich software was used and top 6 results were presented by histogram. In addition, the GeneAnalytics database (<https://ga.genecards.org/>) was used to identify the pathway of the few significant genes eventually identified. And top 10 pathways were downloaded and listed in this study.

***Protein-protein interaction (PPI) network and hub gene identification***

By the using of STRING database (<https://cn.string-db.org/>), the network of gene and protein interactions were analyzed. Confidence score 0.4 was set. Cytoscape software (v3.9.1) was used for the visualization of the data downloaded from STRING database. Sub networks were identified by MCODE app. And the top 10 hub genes were identified by cytoHubba app used MCC method.

***Inference score of hub genes on different diseases***

CTDbase (<http://ctdbase.org>) was used to search the inference score of hub genes on osteoporosis, osteoarthritis and musculoskeletal abnormalities. Then the scores was visualized via a histogram by R software.

***Gene-miRNA, gene-disease and signaling networks analysis***

The database NetworkAnalyst database (<https://www.networkanalyst.ca/>) was used to analyze the gene-miRNA and gene-disease of the DEGs. And the gene-pathway of the significant genes eventually identified was also researched by this database. “H. sapiens (human)” was selected in the “specify organism” column, and the “set ID type” column was set as “official gene symbol”. After uploading genes, the “Gene-miRNA Interactions”, “Gene-disease Associations” and the “Signaling Network” were chosen respectively. Still the “Minimum Network” was also chosen for gene-miRNA and gene-disease network.

***Immunohistochemistry and immunofluorescence of the significant genes from HPA database***

In this step, the human protein atlas (HPA, <https://www.proteinatlas.org/>) was utilized to visualize the immunohistochemistry and immunofluorescence of the significant genes. Bone marrow immunohistochemistry, skeletal muscle immunohistochemistry (because there were no bone tissue immunohistochemistry results of the significant genes in HPA, skeletal muscle results were chosen) and the positive immunofluorescence results in cells were searched and exhibited. And the intracellular localizations were also identified by HPA database.

***Single cell sequencing data analysis from GEO database***

GSE147287 (single cell sequencing data of osteoporosis and osteoarthritis patients, GPL24676) was downloaded from GEO database [5]. Seurat package (V4.3.0) of R software was used to undertake single cell analysis [6]. We set minGene = 200, maxGen e= 5000, pctMT = 5 and dims = 1:20. Single Rpakage (V2.0) was used to identify the cell type of each cluster [7]. In order to do pseudotime analysis, monocle package (V2.26.0) was applied.

***Chemicals and Reagents***

Rat primary BMSC cells were acquired from Institute of Orthopedics, Soochow University, Soochow, China. Thermo Fisher Scientific (St. Louis, USA) provided ɑ-MEM and fetal bovine serum (FBS) for culturing BMSCs, as well as phosphate buffer saline (PBS). Antibodies of anti-VEGFA were obtained from Abclonal, Wuhan, China. The osteogenic inducing medium was obtained from Amizona, Hangzhou, China.

***Cell culture***

Rat primary BMSC cells were acquired from Institute of Orthopedics, Soochow University and cultured in ɑ-MEM medium supplemented with 10% fetal bovine serum. The cells were cultured under 37℃ with 5% CO2 and cells from passages 3-5 were selected for further use. After planting attaching to the plate, cells were cultured with osteogenic inductive medium for inducing osteogenesis.

***Real-time PCR***

Total RNA from BMSCs after 3 day’s inducing was obtained by a TRIzol reagent (Beyotime, China) and quantified with a NanoDrop 2000 system of Thermo Fisher Scientific. Reverse transcription was undertaken to get cDNA from the isolated RNA. A reaction system of 10 μl of qPCR Master Mix, 0.5 μl of forward and reverse primers, 2 μl of cDNA, and 7 μl of nuclease-free ddH2O was used. The comparative 2^−ΔΔCq^ method was applied to analyzed the folding changes of mRNA expression level. The gene primer sequences used in this study are listed in Table 1.

***Immunofluorescence assay***

After 3 day’s inducing, the cells were fixed with 4% paraformaldehyde and 0.2% Triton X-100 (Beyotime, China) on ice. And then QuickBlock buffer (Beyotime, China) was used to blocked the BMSCs for about one hour. Cells were incubated with primary antibodies against VEGFA overnight at 4 °C. F-actin (Yeasen, China) and DAPI (Beyotime, China) were used for a duration of 10 minutes. Cells were then observed and photographed by a fluorescence microscope (Zeiss, Germany).

***Alizarin red staining***

BMSCs were induced osteogenesis for about 21 days. Cells were fixed with 4% paraformaldehyde for about 10 minutes and then washed by ddH_2_O. Then Alizarin red staining S kit (Solarbio, Beijing, China) was used to incubate for about 30 minutes and followed by washing with ddH2O. Cells were photographed by a microscope at last (Zeiss, Germany).

1. Benisch P, Schilling T, Klein-Hitpass L, Frey SP, Seefried L, Raaijmakers N, et al. The transcriptional profile of mesenchymal stem cell populations in primary osteoporosis is distinct and shows overexpression of osteogenic inhibitors. PLoS One. 2012;7(9):e45142. Epub 20120924. doi: 10.1371/journal.pone.0045142. PubMed PMID: 23028809; PubMed Central PMCID: PMCPMC3454401.

2. Sathiyanathan P, Samsonraj RM, Tan CLL, Ling L, Lezhava A, Nurcombe V, et al. A genomic biomarker that identifies human bone marrow-derived mesenchymal stem cells with high scalability. Stem Cells. 2020;38(9):1124-36. Epub 20200608. doi: 10.1002/stem.3203. PubMed PMID: 32510174.

3. Zheng H, Liu J, Yu J, McAlinden A. Expression profiling of mitochondria-associated microRNAs during osteogenic differentiation of human MSCs. Bone. 2021;151:116058. Epub 20210615. doi: 10.1016/j.bone.2021.116058. PubMed PMID: 34144232; PubMed Central PMCID: PMCPMC8944210.

4. Zhou Y, Zhou B, Pache L, Chang M, Khodabakhshi AH, Tanaseichuk O, et al. Metascape provides a biologist-oriented resource for the analysis of systems-level datasets. Nat Commun. 2019;10(1):1523. Epub 20190403. doi: 10.1038/s41467-019-09234-6. PubMed PMID: 30944313; PubMed Central PMCID: PMCPMC6447622.

5. Wang Z, Li X, Yang J, Gong Y, Zhang H, Qiu X, et al. Single-cell RNA sequencing deconvolutes the in vivo heterogeneity of human bone marrow-derived mesenchymal stem cells. Int J Biol Sci. 2021;17(15):4192-206. doi: 10.7150/ijbs.61950. PubMed PMID: 34803492; PubMed Central PMCID: PMCQ1.

6. Hao Y, Hao S, Andersen-Nissen E, Mauck WM, Zheng S, Butler A, et al. Integrated analysis of multimodal single-cell data. Cell. 2021;184(13). doi: 10.1016/j.cell.2021.04.048. PubMed PMID: 34062119; PubMed Central PMCID: PMCQ1.

7. Aran D, Looney AP, Liu L, Wu E, Fong V, Hsu A, et al. Reference-based analysis of lung single-cell sequencing reveals a transitional profibrotic macrophage. Nat Immunol. 2019;20(2):163-72. doi: 10.1038/s41590-018-0276-y. PubMed PMID: 30643263; PubMed Central PMCID: PMCQ1.
