# Supplementary material for: Identification of ferroptosis/autophagy-related genes and potential underlying mechanisms involved in the effect of BMSC senescence on the osteogenic differentiation of aging BMSCs
Source: Genes Dis. 2024 Mar 8;12(1):101259. doi: 10.1016/j.gendis.2024.101259 (PMC11530586; doi:10.1016/j.gendis.2024.101259)
Supplement: Multimedia component 2 [file mmc2.doc]

Supplementary Table 1. Primers used in qRT-PCR.

| Gene | Primer sequence (F) | Primer sequence (R) |
| --- | --- | --- |
| ATG9A | CCGAGGTGCTGAAGAGAGA | TACTTGGAGGCGGGCTT |
| VEGFA | ACAGGGAAGACAATGGGA | CTGGAAGTGAGCCAACG |
| MAPK14 | GGGGCAGACCTGAACAAC | AACTGAACGTGGTCATCGG |
| SLC2A1 | AGGCTTTACCGCAGGAG | AGTGGTTGGTTGAGTTGGA |
| GAPDH | ATGGCTACAGCAACAGGGT | TTATGGGGTCTGGGATGG |

Supplementary Table 2. Gene distribution and corresponding miRNAs of the 40 genes.

| The 40 genes | expression | autophagy | ferroptosis | GSE35957 | GSE135401 | corresponding differentially expressed miRNAs in GSE134946 |
| --- | --- | --- | --- | --- | --- | --- |
| **ATG9A** | **up** | **yes** |  |  | **yes** | **hsa-miR-34c-5p; hsa-miR-15b-5p** |
| BCL2 | down | yes |  |  | yes | hsa-miR-34c-5p; hsa-miR-204-5p; hsa-miR-202-5p; hsa-miR-15b-5p |
| EPG5 | up | yes |  | yes |  | hsa-miR-30a-5p |
| CANX | up | yes |  | yes |  | hsa-miR-148a-3p |
| CTNS | up | yes |  | yes |  | hsa-let-7d-5p |
| NCKAP1 | up | yes |  | yes |  | hsa-miR-520f-3p |
| MAP2K7 | up | yes |  | yes |  | hsa-miR-125b-5p; hsa-miR-125a-5p |
| DAPK2 | down | yes |  | yes |  | hsa-miR-133a-3p |
| VAMP8 | down | yes |  | yes |  | hsa-miR-103a-3p; hsa-miR-15b-5p |
| EIF4EBP1 | down | yes |  | yes |  | hsa-miR-125b-5p; hsa-miR-328-3p; hsa-miR-125a-5p |
| NBR1 | down | yes |  | yes |  | hsa-miR-15b-5p |
| ITPR1 | down | yes |  | yes |  | hsa-miR-92b-3p |
| GABARAPL1 | up | yes | yes | yes |  | hsa-miR-133a-3p; hsa-miR-15b-5p |
| EGFR | up | yes | yes | yes |  | hsa-miR-148a-3p |
| LAMP2 | up | yes | yes | yes |  | hsa-miR-193b-3p |
| **VEGFA** | **down** | **yes** | **yes** | **yes** |  | **hsa-miR-15b-5p** |
| TFAP2A | up |  | yes |  | yes | hsa-miR-133a-3p |
| PANX2 | up |  | yes |  | yes | hsa-let-7d-5p |
| TNFAIP3 | up |  | yes |  | yes | hsa-miR-125b-5p; hsa-miR-125a-5p |
| MAP3K5 | down |  | yes |  | yes | hsa-miR-30a-5p |
| **MAPK14** | **down** |  | **yes** |  | **yes** | **hsa-miR-125b-5p; hsa-miR-125a-5p** |
| NF2 | down |  | yes |  | yes | hsa-miR-92b-3p |
| RGS4 | up |  | yes | yes | yes | hsa-miR-103a-3p |
| HELLS | down |  | yes | yes | yes | hsa-miR-103a-3p; hsa-miR-204-5p |
| GCLC | up |  | yes | yes |  | hsa-miR-30a-5p; hsa-miR-133a-3p |
| GCH1 | up |  | yes | yes |  | hsa-miR-34c-5p; hsa-miR-133a-3p |
| PRKAA2 | up |  | yes | yes |  | hsa-let-7d-5p |
| DDIT4 | down |  | yes | yes |  | hsa-miR-30a-5p |
| MAFG | down |  | yes | yes |  | hsa-miR-148a-3p; hsa-miR-204-5p |
| **SLC2A1** | **down** |  | **yes** | **yes** |  | **hsa-miR-148a-3p; hsa-miR-328-3p** |
| SLC7A5 | down |  | yes | yes |  | hsa-miR-148a-3p |
| RRM2 | down |  | yes | yes |  | hsa-let-7d-5p |
| SCD | down |  | yes | yes |  | hsa-let-7d-5p |
| SLC1A4 | down |  | yes | yes |  | hsa-let-7d-5p |
| LURAP1L | down |  | yes | yes |  | hsa-miR-381-3p; hsa-miR-125b-5p; hsa-miR-15b-5p; hsa-miR-92b-3p; hsa-miR-125a-5p |
| EPT1 | down |  | yes | yes |  | hsa-miR-15b-5p |
| FLT3 | down |  | yes | yes |  | hsa-miR-15b-5p |
| GLS2 | down |  | yes | yes |  | hsa-miR-15b-5p |
| AURKA | down |  | yes | yes |  | hsa-miR-92b-3p |
| FANCD2 | down |  | yes | yes |  | hsa-miR-193a-5p |

Supplementary Table 3. Top 10 pathways of the 4 genes identified by GeneAnalytics database.

| pathway | score | gene |
| --- | --- | --- |
| Angiogenesis (WikiPathways) | 17.08 | VEGFA, MAPK14 |
| HIF-2-alpha Transcription Factor Network | 16.08 | VEGFA, SLC2A1 |
| Photodynamic Therapy-induced HIF-1 Survival Signaling | 15.83 | VEGFA, SLC2A1 |
| S1P3 Pathway | 15.08 | VEGFA, MAPK14 |
| Cell Adhesion_Plasmin Signaling | 15.02 | VEGFA, MAPK14 |
| Hepatitis C and Hepatocellular Carcinoma | 14.91 | VEGFA, MAPK14 |
| **Cellular Senescence** | **14.81** | **VEGFA, MAPK14, ATG9A** |
| HIF1Alpha Pathway | 14.74 | VEGFA, SLC2A1 |
| HIF-1-alpha Transcription Factor Network | 14.21 | VEGFA, SLC2A1 |
| Neural Stem Cells and Lineage-specific Markers | 13.65 | VEGFA, SLC2A1 |
