# Supplementary material for: Identification of ferroptosis/autophagy-related genes and potential underlying mechanisms involved in the effect of BMSC senescence on the osteogenic differentiation of aging BMSCs
Source: Genes Dis. 2024 Mar 8;12(1):101259. doi: 10.1016/j.gendis.2024.101259 (PMC11530586; doi:10.1016/j.gendis.2024.101259)
Supplement: Multimedia component 3 [file mmc3.pptx]

## Slide 1
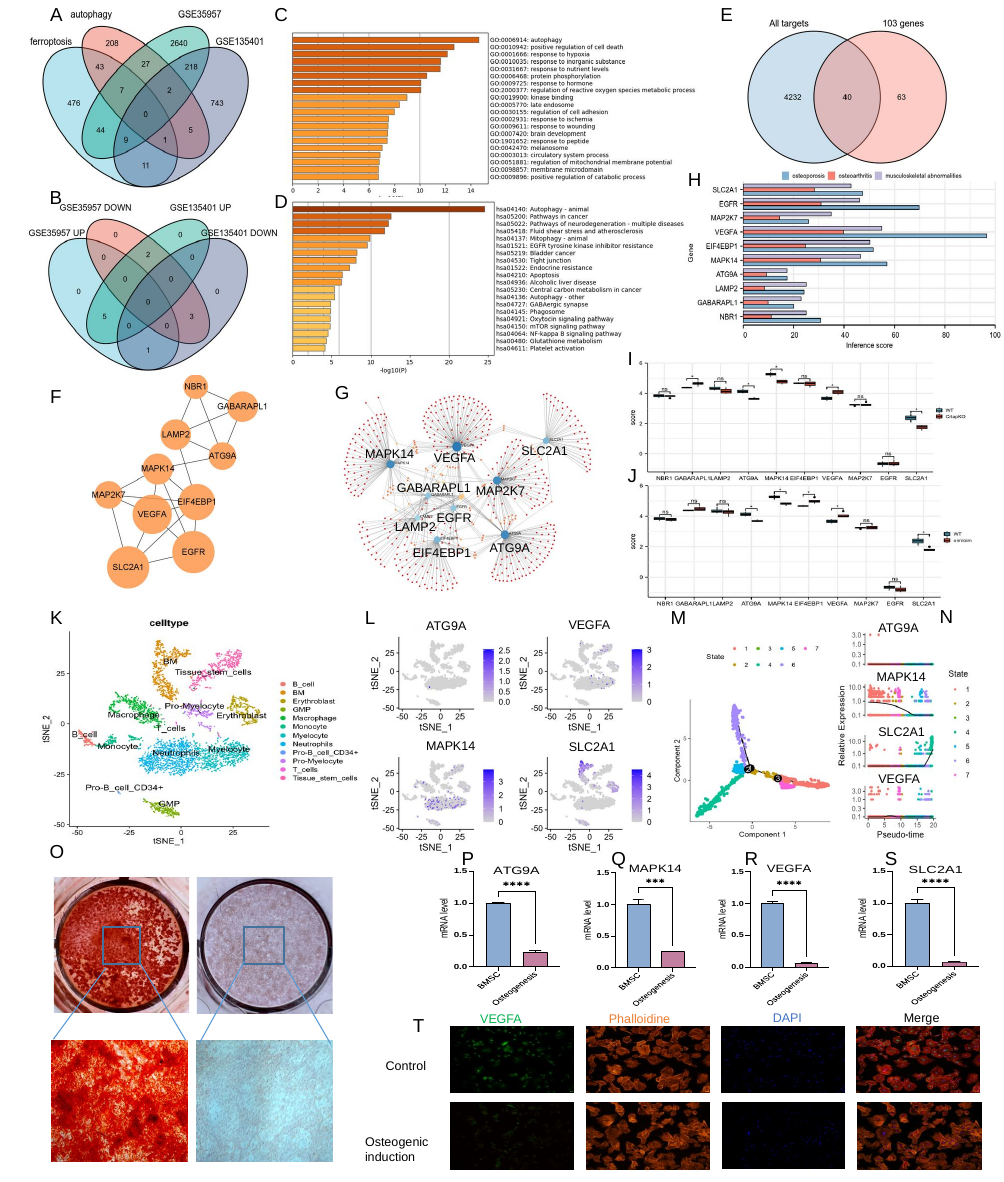

A
C
E
H
B
D
I
G
F
SLC2A1
MAPK14
VEGFA
J
GABARAPL1
MAP2K7
EGFR
LAMP2
ATG9A
EIF4EBP1
N
K
L
M
VEGFA
ATG9A
MAPK14
SLC2A1
ATG9A
MAPK14
SLC2A1
VEGFA
O
P
Q
R
S
Merge
DAPI
VEGFA
Phalloidine
Control
Osteogenic induction
T

## Slide 2
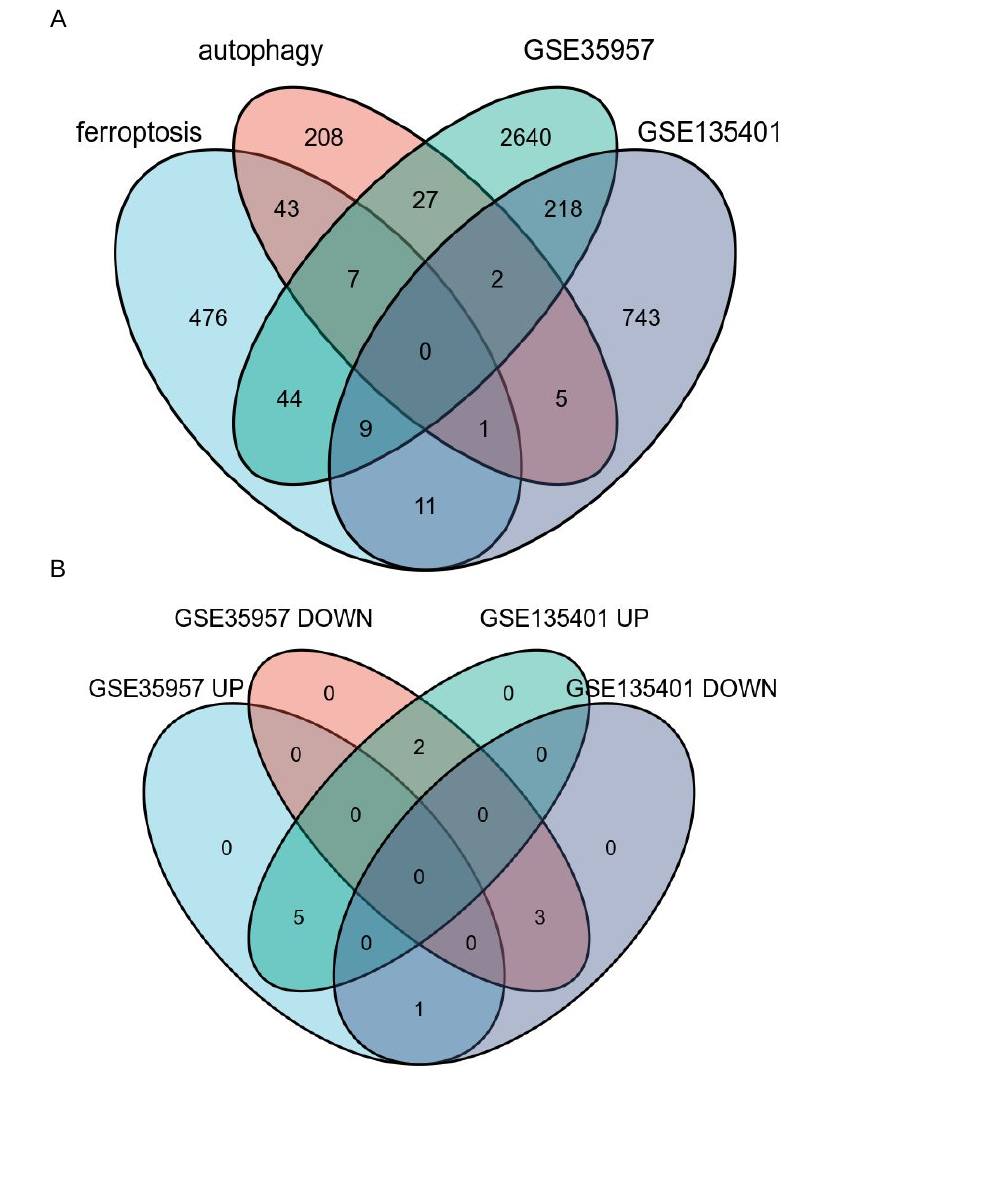

A
B

## Slide 3
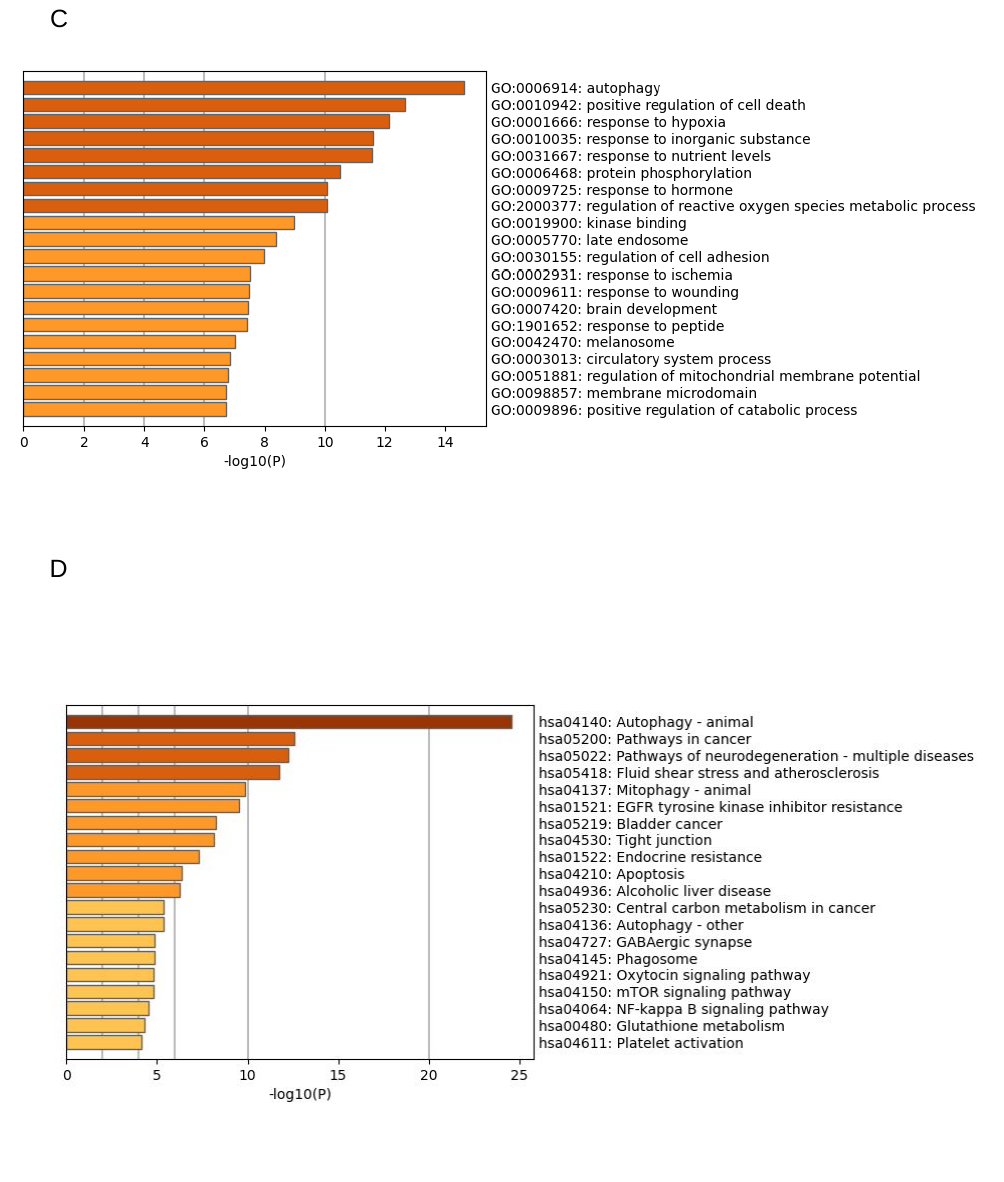

C
D

## Slide 4
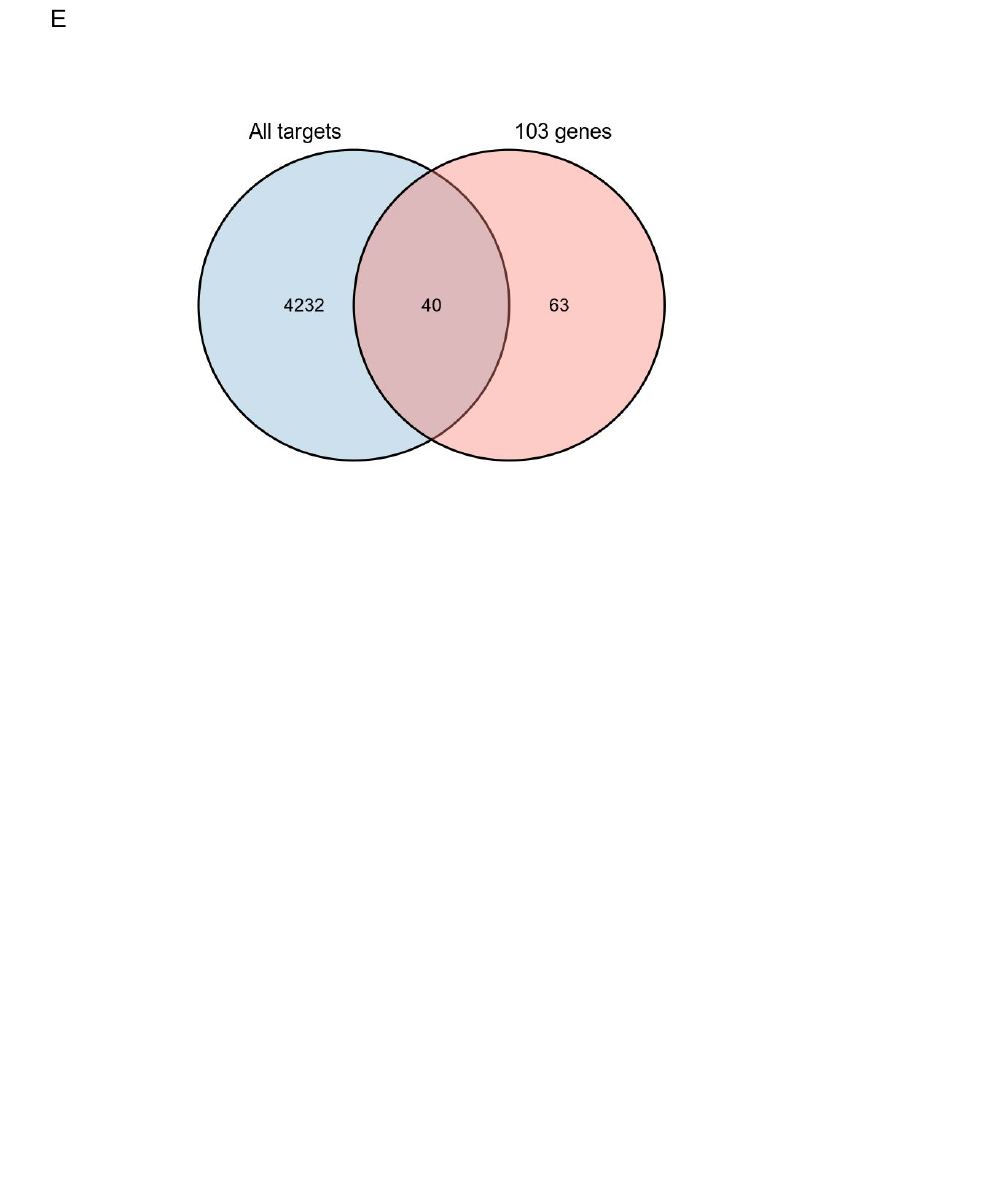

E

## Slide 5
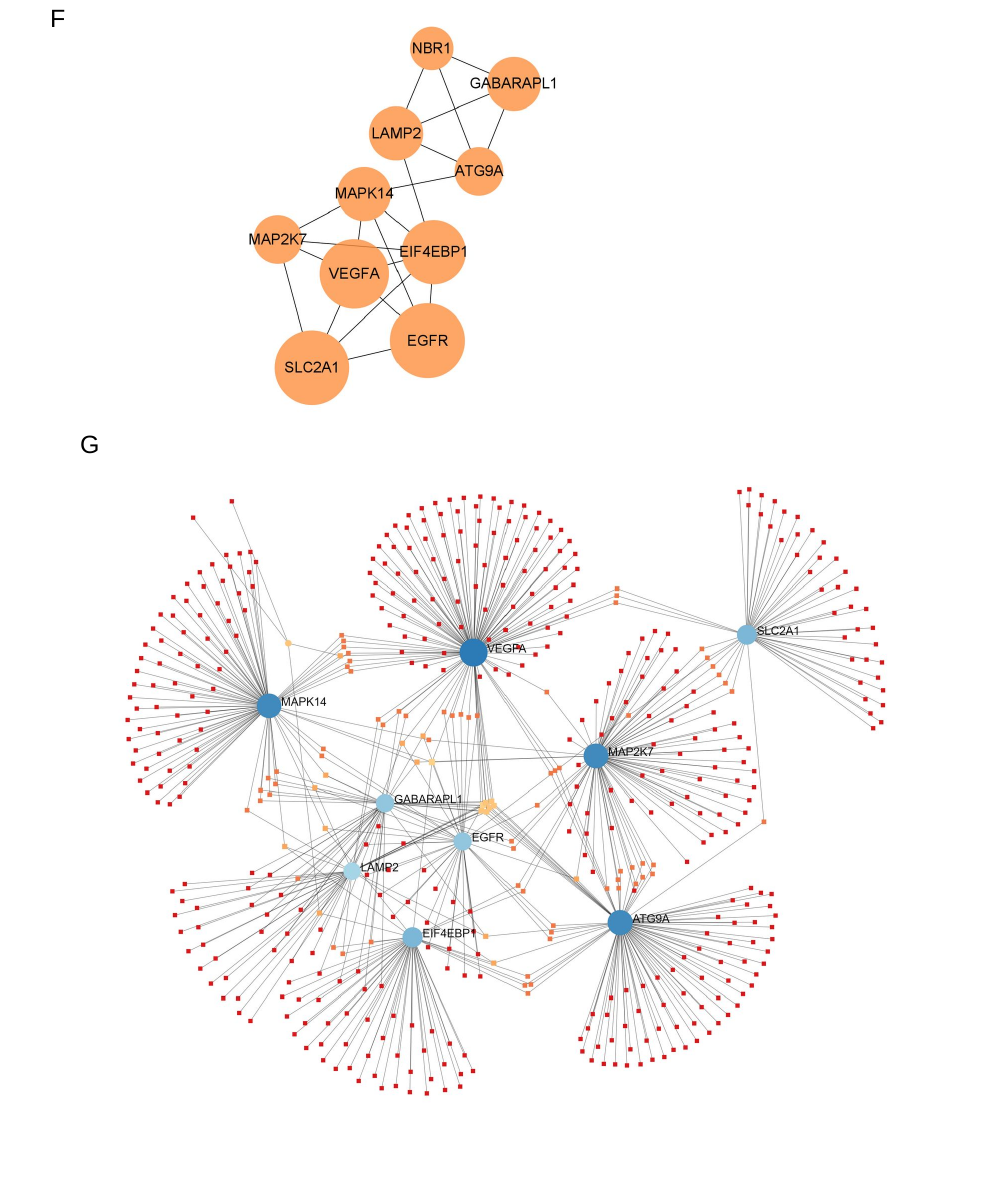

F
G

## Slide 6
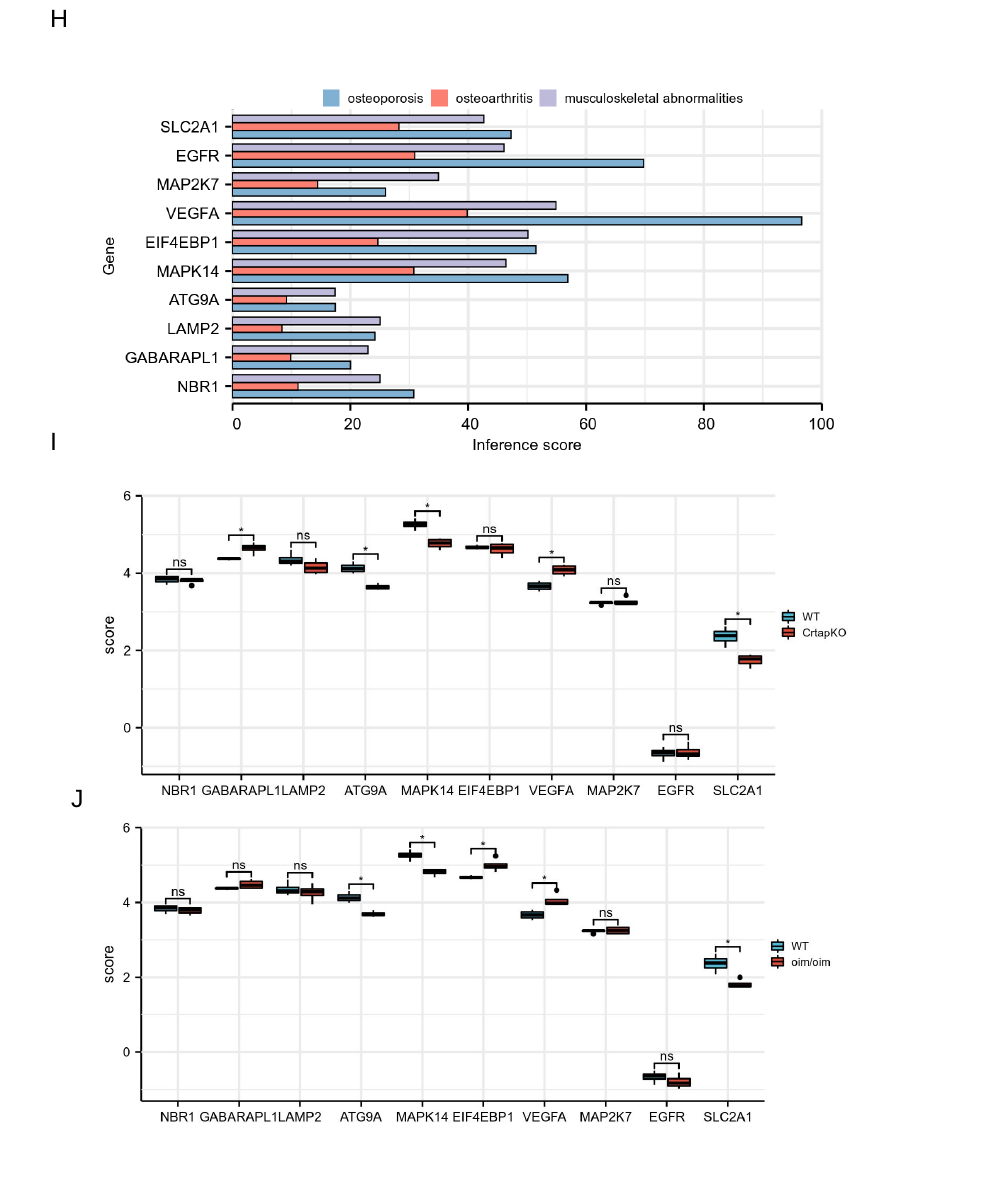

H
I
J

## Slide 7
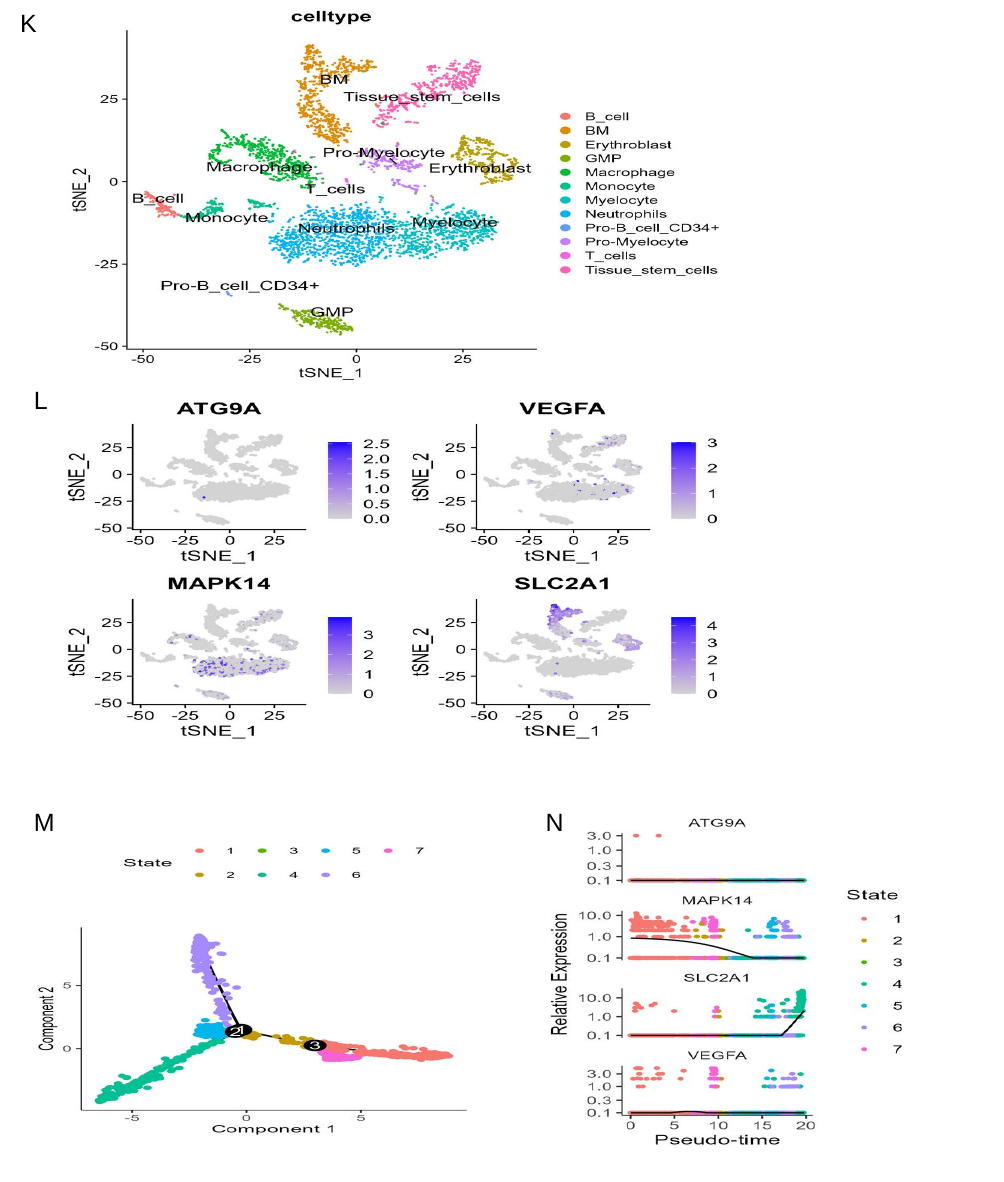

K
L
M
N

## Slide 8
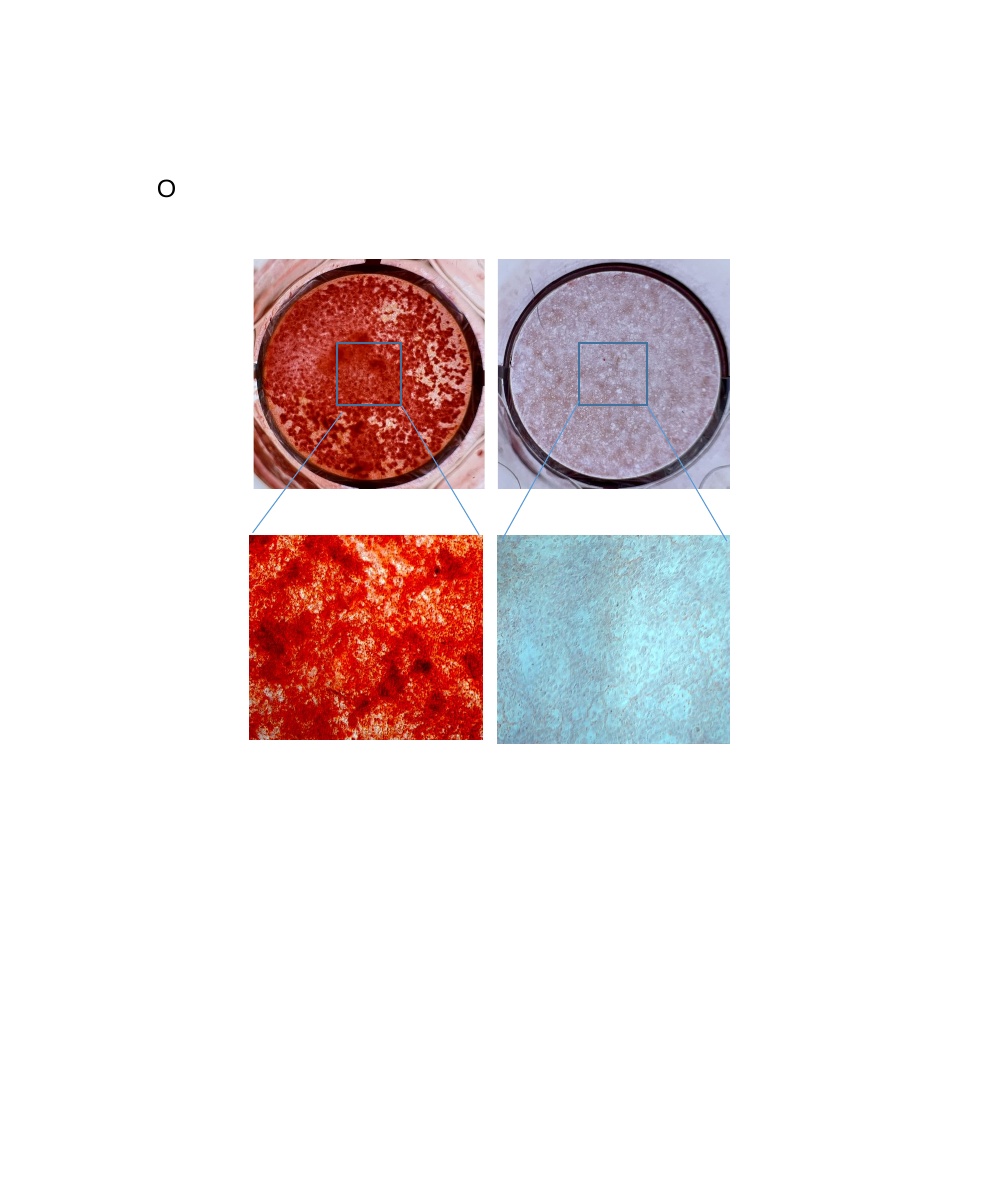

O

## Slide 9
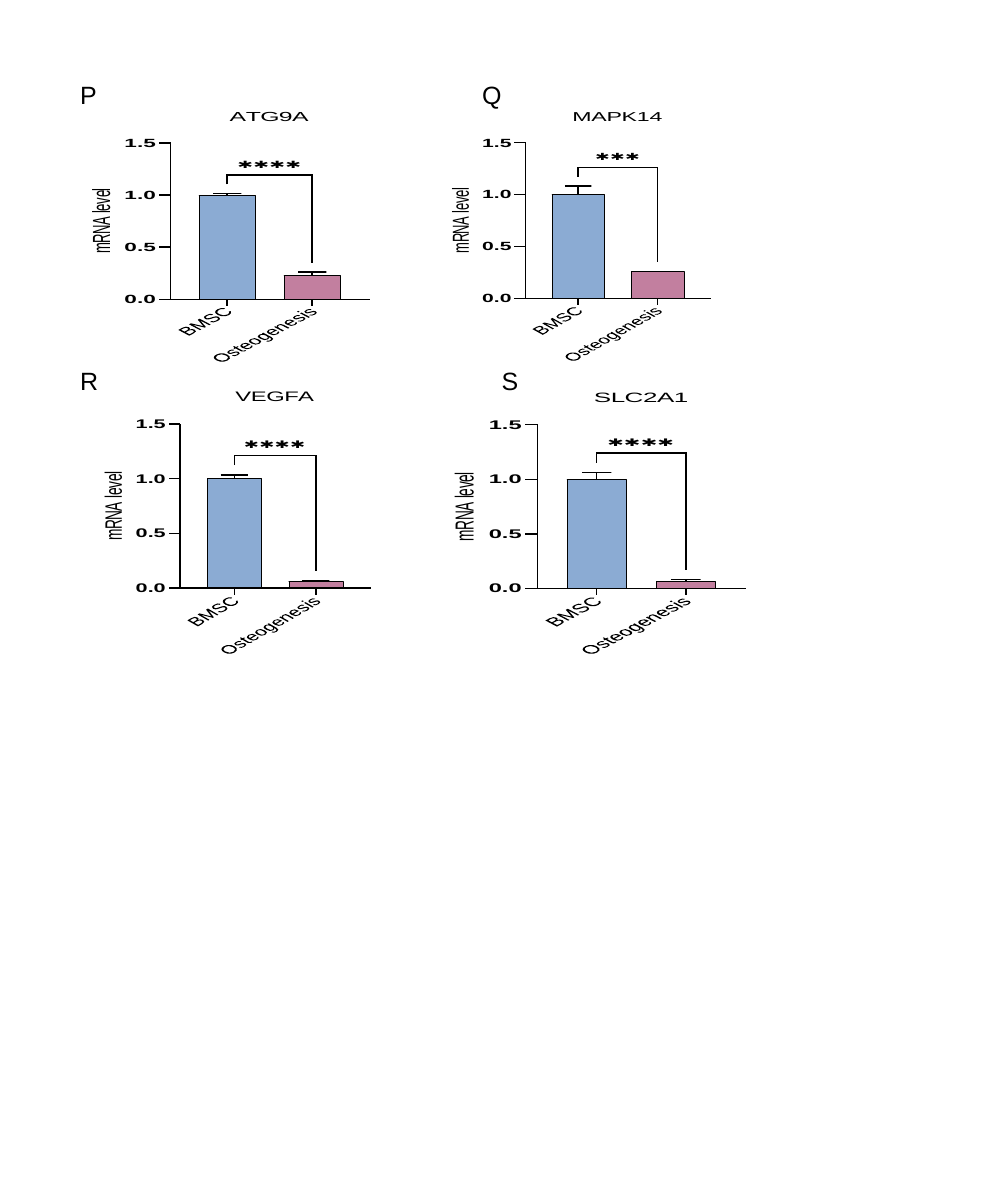

P
Q
R
S

## Slide 10
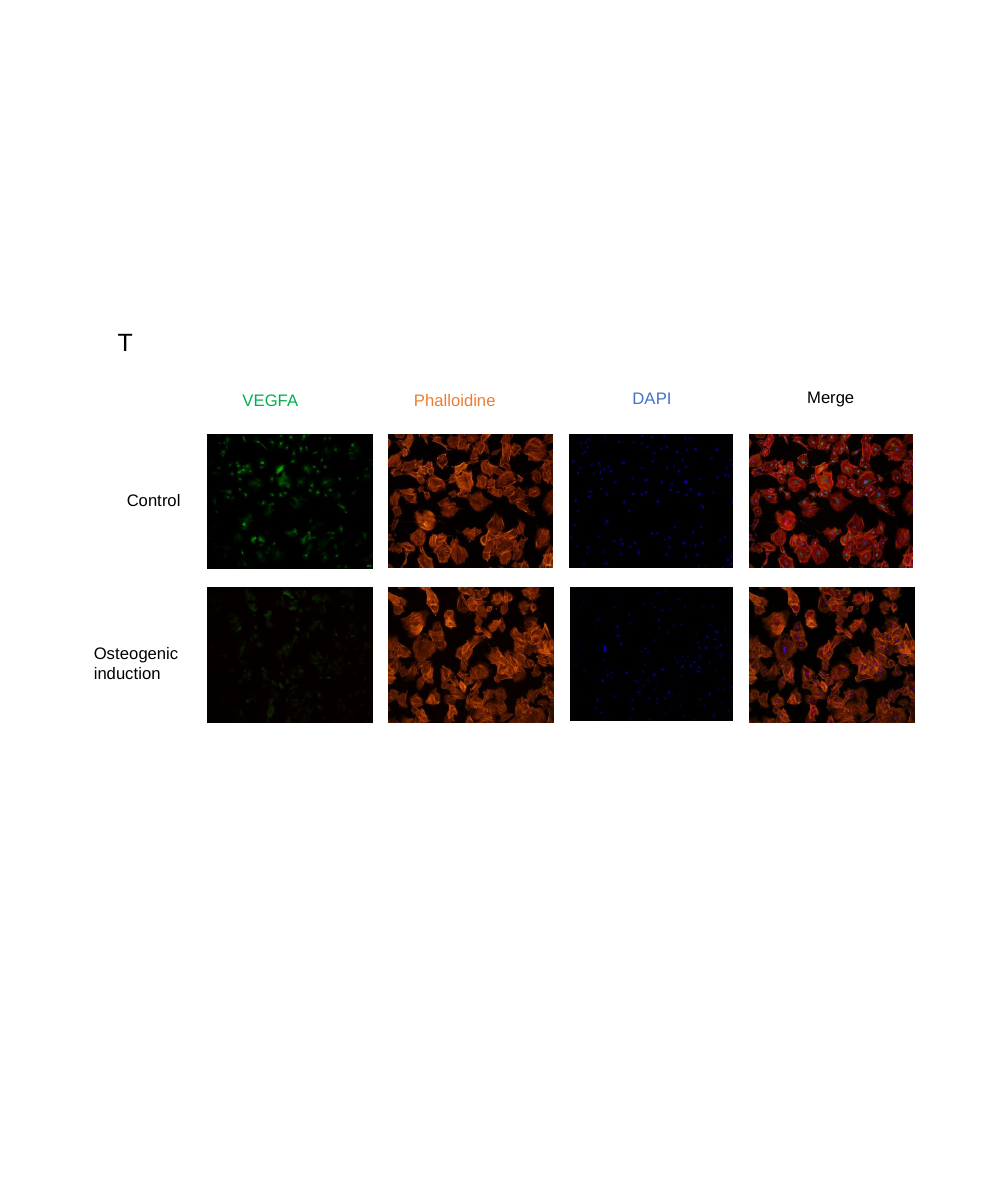

T
Merge
DAPI
VEGFA
Phalloidine
Control
Osteogenic induction
